# Exploring non-compliance with traffic laws and its role in road crashes in Yazd: a qualitative content analysis

**DOI:** 10.1186/s13104-026-07732-7

**Published:** 2026-03-09

**Authors:** Maryam Ghavami, SeyedSaeed MazloomyMahmoodabad, Shakiba Zahed

**Affiliations:** 1 Social Determinants of Health Research Center, Department Health Education and Promotion, School of Public Health, Shahid Sadoughi university of medical sciences, Yazd, Iran; 2 Health Education and Health Promotion, Social Determinants of Health Research Center, Non-communicable Diseases Research Institute, Department of Health Education and Promotion, School of Public Health, Shahid Sadoughi University of Medical Sciences, Yazd, Iran; 3 Health Education and Health Promotion - Isfahan university of medical sciences, Isfahan, Iran

**Keywords:** Road traffic crashes, Non-compliance, Motorcycling, Qualitative content analysis, Yazd, Iran

## Abstract

**Objective:**

This qualitative content analysis aimed to explore non-compliance with traffic laws and its perceived contribution to road traffic crashes in Yazd, with particular emphasis on motorcycling, from the perspectives of stakeholders.

**Results:**

Conventional qualitative content analysis of semi-structured in-depth interviews with 14 purposively sampled participants identified three main themes: (1) operational policies, including deficient urban infrastructure (such as narrow streets and lack of pedestrian facilities) and weak law enforcement; (2) human factors, such as low compliance culture, personality traits (including impatience and thrill-seeking), and residency-related differences; and (3) educational challenges, particularly inadequate initial and periodic driver training. Participants emphasized that these interacting elements, especially poor infrastructure and insufficient enforcement (notably toward motorcyclists), substantially increase crash risk in this setting.

## Background

Road traffic crashes are a leading cause of mortality and disability worldwide, with approximately 1.19 million deaths and up to 50 million injuries annually, disproportionately affecting low- and middle-income countries (LMICs) [1, 2]. In Iran, road traffic fatalities average 22,185 per year, primarily involving young people on suburban roads [3, 4]. Human factors such as speeding, non-use of seatbelts, and inadequate following distance, combined with environmental and social influences, are well-established contributors [5, 6].

In Yazd Province, crash rates are twice the national average, with Azadshahr—a southwestern district characterized by rapid urban growth, a high migrant population, and infrastructural constraints—experiencing particularly high rates (approximately 2.5–3 times the city-wide average) [7]. According to the Yazd Province Traffic Police (Mehr News Agency, 2023), areas such as Azadegan Boulevard and Azadi Square are among the top urban black spots in the province. Motorcyclists and pedestrians are especially vulnerable, and non-compliance with traffic laws is consistently identified as a major driver of crash frequency and severity.

Although quantitative studies predominate in the Iranian literature, few have used qualitative methods to explore residents’ lived experiences and perceptions in high-risk urban neighborhoods. This study, conducted in collaboration with Shahid Sadoughi University’s School of Public Health, local law enforcement, and provincial stakeholders, employed conventional content analysis to investigate contextual factors underlying traffic law non-compliance in Azadshahr, Yazd. The aim was to generate evidence-based, locally relevant recommendations for improving road safety and quality of life.

### Significance and contributions of the study

This study provides the first qualitative exploration of traffic law non-compliance in Azadshahr, a district marked by rapid urbanization, migrant influx, and severe infrastructural constraints. By capturing residents’ perspectives, it addresses a critical evidence gap that quantitative approaches alone cannot fill. Key contributions include: identification of three interconnected themes (operational policies, human factors, and educational challenges) offering a comprehensive socio-ecological framework absent from prior Iranian traffic safety research; generation of actionable, context-specific recommendations (e.g., infrastructure upgrades, targeted enforcement, and ongoing driver education); advancement of qualitative methodology in road safety studies within LMICs; and direct support for Sustainable Development Goal 3.6 (halving global road traffic deaths by 2030) through health-promotion-oriented strategies [8].

## Methods

This qualitative study employed a conventional content analysis approach as described by Graneheim and Lundman [9], adapted to examine traffic-related behaviors. The study was conducted between 2023 and 2024 following ethical approval (code: IR.SSU.SPH.REC.1402.176) from Shahid Sadoughi University of Medical Sciences, Yazd, Iran.

*Inclusion criteria:* Eligible participants were literate adults aged 18 years or older, residing in or present in Azadshahr, Yazd, who possessed a valid driver’s license (for drivers) or were actively using the area as pedestrians, demonstrated effective communication skills, and voluntarily agreed to share their experiences related to traffic law compliance and road crashes.

*Participant selection:* Purposive sampling with maximum variation was used to recruit 14 participants, ensuring diversity in gender, age, educational level, socioeconomic status, driving experience (private cars, commercial vehicles, public transport, motorcycles), and pedestrian status. Drivers were selected based on their active driving experience in Yazd city, while pedestrians were interviewed in their role as non-vehicle users at the time of the interview to capture authentic lived experiences. Recruitment occurred in varied settings, including streets, alleys, law enforcement offices, and traffic departments. To preserve confidentiality, participants were described only in general categories without personal identifiers.

*Data Collection and Analysis;* Data were collected via semi-structured in-depth interviews (lasting 20–60 min) and systematic field observations in key traffic locations (e.g., roundabouts, boulevards, intersections) to capture contextual non-compliance behaviors. Interview schedules and locations were participant-determined. Each session began with gratitude, study explanation, and explicit consent for audio recording; participants could stop recording at any time.

The interview guide, developed based on methodological recommendations [10], included core questions such as: “Please describe your experience and the cause of your crash,” “What do you think causes the high number of crashes in this area?” “Have you ever had or witnessed a traffic crash?” and “What is your opinion on compliance with traffic regulations?” Demographic information (age, education, marital status, crash/injury history) was documented.

Data collection continued until thematic saturation was reached, with no new codes emerging. Interviews were audio-recorded, transcribed verbatim, and analyzed line-by-line using Microsoft Word’s comment feature to identify initial codes. Codes were grouped iteratively into categories and themes following Graneheim and Lundman’s approach [9]. Triangulation was achieved by integrating field observation notes with interview data to enhance credibility. Trustworthiness was ensured using Lincoln and Guba’s criteria through prolonged engagement with diverse participants, member checking, peer debriefing, inter-coder agreement (kappa = 0.82), and external audit [11].

**Table 1:** Demographic characteristics of participants

Variable	Indicator	Frequency (*n* = 14)	Percentage (%)
Gender	Man	12	85.71
	Female	2	14.29
Age	18–29 years	3	21.42
	30–59 years	11	78.58
	≥ 60 years	0	0
Education	High school	1	7
	Diploma	6	43
	University degree	7	50
Crash History	Has crash history	6	43
	No crash history	8	57
Injury History	Has injury history	1	7
	No injury history	13	93
Occupation	Unemployed	0	0
	Homemaker	1	7
	Employee	3	21
	Self-employed	10	72
	Retired	0	0
Total	–	14	100

**Table 2:** Themes, Subcategories, and specific details

Main Category	Subcategory	Sub-subcategory	Frequency of Mentions (*n* = 14)	Specific Details
Operational Policies	Urban Infrastructure	Narrow Streets	10/14	Leads to congestion and crashes; 20% increase at San’at Roundabout
		Lack of Overpasses	8/14	Unsafe crossings by young motorcyclists in residential areas
		Roundabouts versus Signalized Intersections	7/14	Heightens crashes in close roundabouts (San’at & Azadshahr, 200 m apart)
		Absence of Speed Bumps	9/14	Encourages speeding (160–170 km/h in Tashrifat Blvd)
		Insufficient Street Lighting	6/14	Elevates nighttime risks (Tashrifat → Imam Ali Roundabout)
		Uneven Intersections	5/14	Reduces control at bus stops with narrow pathways
	Traffic Laws & Regulations	Lack of Penalties for Motorcyclists	11/14	Drives reckless behavior, notably in “Alley of Shadows”
		Absence of Seatbelt Penalties	8/14	Increases fatalities at key intersections
		Insufficient Police Presence	9/14	Weakens oversight; linked to fatigue in high-traffic zones
Human Factors	Cultural Factors		7/14	Low compliance culture, prevalent among youth in migrant areas
	Personality Factors		10/14	Impatience and thrill-seeking, especially among drivers under 30
	Residency of non-locals		6/14	Cultural differences increase risks in migrant neighborhoods
Educational Challenges	Lack of Periodic Training		8/14	Reduces awareness of updated regulations (e.g., icy roads)
	Inadequate Licensing Education		9/14	Leaves drivers unprepared for adverse conditions (e.g.,

## Results

**Fig. 1:**
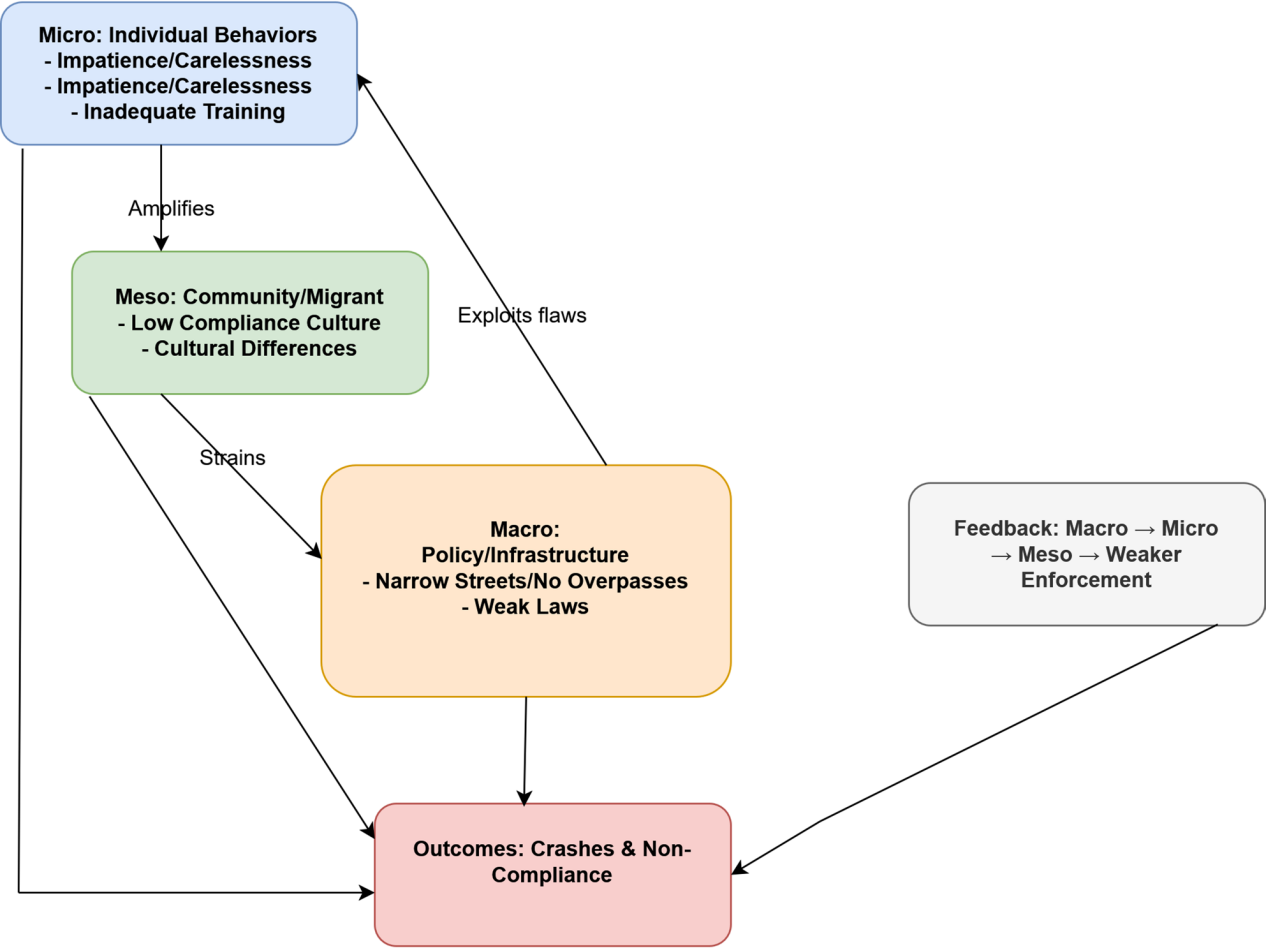
Socio-Ecological Conceptual Model of Traffic Law Non-Compliance

**Fig. 2:**
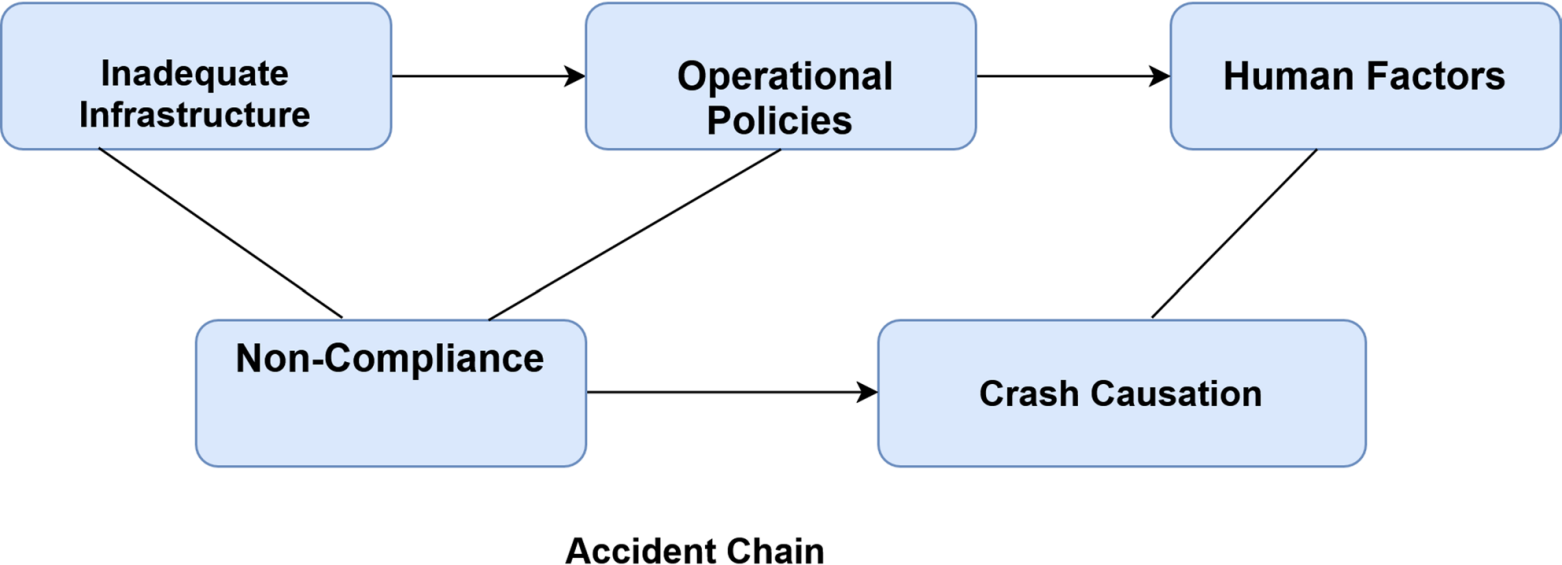
Conceptual model of factors influencing crash causation

This qualitative study in Azadshahr, Yazd, involving 14 participants (2 women, 12 men; mean age 57.73 ± 9.7 years) (Table 1), identified multiple factors contributing to non-compliance with traffic regulations and road crashes through interviews and field observations. The findings were organized into three main themes with their subcategories (see Table 2). Figure 1 presents a socio-ecological conceptual model of traffic law non-compliance, while Fig. 2 illustrates the interrelationships among factors contributing to crash causation, highlighting perceived causal pathways derived from participant narratives and field observations.

1. * Operational Policies*: This theme captured participants’ descriptions of infrastructural and regulatory shortcomings perceived to contribute to non-compliance.
  *Urban infrastructure:* Participants frequently identified deficiencies in urban infrastructure as major contributors to non-compliance and increased crash risk. These included narrow and uneven streets, lack of pedestrian overpasses, hazardous roundabout configurations (e.g., closely spaced roundabouts), absence of speed bumps, insufficient street lighting, and poorly designed intersections. Such issues were perceived to cause congestion, unsafe pedestrian crossings, excessive speeding, and elevated nighttime risks, particularly in locations such as Tashrifat Boulevard, San’at Roundabout, and Imam Ali Roundabout (see Fig. 2 for interrelationships and Table 2 for details and frequencies).
  *Traffic laws and regulations:* Weak enforcement was consistently described as exacerbating non-compliance. Key concerns included lack of penalties for motorcyclists (e.g., riding without helmets or licenses), non-use of seatbelts, and insufficient police presence at critical intersections and high-risk areas (e.g., “Alley of Shadows”). Participants emphasized that stronger supervision and penalties are essential to reduce reckless behaviors (see Table 2; Fig. 2).
2. *Human Factors*: Cultural norms of low compliance, personality traits such as impatience and thrill-seeking (especially among younger drivers), and differences related to residency/migration status were frequently linked to unsafe behaviors and higher crash rates in migrant-heavy areas (see Table 2).
3. *Educational Challenges*: Participants highlighted inadequate initial driver licensing education and the complete lack of periodic refresher training, leaving drivers unprepared for adverse conditions such as snow, ice, or nighttime driving (see Table 2; Fig. 2).

## Discussion

This study categorized factors contributing to traffic law non-compliance and road crashes in Azadshahr into three main themes: operational policies, human factors, and educational challenges.

*Operational policies:* Participants perceived inadequate urban infrastructure (narrow streets, lack of overpasses, poor lighting, uneven intersections) and weak enforcement (limited police presence, no penalties for motorcyclists/helmets/seatbelts) as major contributors to congestion and crashes. These align with prior studies in LMICs emphasizing traffic engineering and supervision [12–14]. The socio-ecological model (Fig. 1) and crash causation framework (Fig. 2) offer a comprehensive basis for multi-level interventions aligned with SDG 3.6 [8].

*Human factors:* Cultural norms of low compliance, personality traits (impatience, thrill-seeking among young drivers), and residency differences (migrant populations introducing varied behaviors) were frequently described as exacerbating unsafe practices, consistent with behavioral research gaps in LMICs [15].

*Educational challenges:* Insufficient initial licensing and lack of periodic training leave drivers unprepared for adverse conditions (e.g., ice, night driving), supporting calls for structured, ongoing education programs [16–19].

## Conclusion

This study identified three major domains—operational policies, human factors, and educational challenges, as key contributors to traffic law non-compliance and crashes in Azadshahr, Yazd.

Operational policies: Participants perceived inadequate urban infrastructure and weak enforcement of traffic regulations as significantly contributing to increased crash risks. Improvements in road design, lighting, pedestrian facilities, and stricter enforcement of helmet and seatbelt laws are urgently needed.

Human factors: Cultural norms of low compliance, personality traits such as impatience and thrill-seeking, and residency differences were described by participants as exacerbating unsafe behaviors. Addressing these requires culturally sensitive interventions and community-based awareness programs.

Educational challenges: Insufficient driver training and lack of periodic education were perceived as leaving drivers unprepared for adverse conditions. Structured licensing systems and ongoing training programs can strengthen driver preparedness and reduce crash rates.

Taken together, these findings underscore the necessity of a comprehensive, multi-level approach informed by global evidence and aligned with Sustainable Development Goals (SDGs).

### Policy recommendations

To improve feasibility, recommendations are structured into short- and long-term actions with designated stakeholders, integrating operational, behavioral, and educational reforms while leveraging technologies like mobile apps and virtual reality for safer driving.

### Short-term actions (1–2 years)

Municipal installation of speed bumps and better lighting in high-risk areas (e.g., Tashrifat Boulevard).

Increased police patrols at key spots (e.g., San’at Roundabout) to enforce helmet use among young motorcyclists.

Launch of culturally sensitive awareness campaigns targeting drivers under 30, led by education authorities.

### Long-term strategies (3–5 years)

Municipal-led expansion of infrastructure (e.g., pedestrian overpasses and wider roads) with traffic engineering collaboration.

Development of a multi-stage driver licensing program by transportation and education authorities.

Sustained enforcement via stronger police presence and stricter penalties.

Implementing these reforms will help Azadshahr achieve a safer, more sustainable traffic system, reducing crashes and enhancing public health.

Future Research Recommendation A mixed-methods follow-up study with a larger, stratified sample (*n* ≥ 100) is proposed to quantitatively validate current findings and improve generalizability, combining interviews, surveys, and crash data analysis across diverse demographics.

### Strengths

Comprehensive analysis: Employed qualitative interviews to identify diverse factors contributing to traffic violations. Diverse participants: Incorporated perspectives from individuals of varying ages and professions, including drivers, police officers, and instructors. Precise categorization: Organized factors into clear main categories and subcategories for enhanced problem understanding. Use of conceptual models: The socio-ecological (Fig. 1) and crash causation (Fig. 2) models assist readers in understanding the interrelationships among factors contributing to crashes.

### Limitations

The study acknowledges several limitations. The small sample size of 14 participants (2 women, 12 men), while sufficient for qualitative saturation in a focused context like Azadshahr, limits the breadth of perspectives and restricts quantitative validation, necessitating caution in interpreting statistical robustness. The findings are context-specific, reflecting the unique infrastructural challenges (e.g., narrow streets in San’at Roundabout) and cultural dynamics (e.g., low compliance among migrants) of Azadshahr, Yazd, and are not generalizable to other regions without further validation. Challenges in including foreign nationals due to language barriers and limited access to certain neighborhoods constrained sample representativeness. Environmental and economic factors influencing traffic behavior were not explored, and time/resource constraints may hinder immediate implementation of recommendations. Lastly, the absence of a quantitative risk assessment (e.g., crash frequency-severity index) limits spatial analysis depth, suggesting a need for future mixed-methods research.

Nevertheless, this study opens avenues for future research, including similar qualitative investigations in other urban contexts, mixed-methods approaches integrating crash data, exploration of innovative educational interventions (e.g., simulation training, mobile applications), and longitudinal studies on policy impacts.

## Data Availability

the datasets generated and/or analyzed during the current study are available from the corresponding author on reasonable request.

## Abbreviations

LMICs: Low- and middle-income countries
SDG: Sustainable Development Goal
WHO: World Health Organization

## References

[1] Ahmad A, Yadav P, Pant K, Tripathi A, Dubey G. Reviewing the prevalence of refractive errors and color blindness among commercial drivers. Int J Community Med Public Health. 2024;11(8):3309–13.

[2] Najimi-Varzaneh A, Gholami Fesharaki M. Prevalence of road traffic accidents in iran: a systematic review, GIS and meta-analysis. Iran Red Crescent Med J. 2018;20(10):e83852.

[3] Mazloomy Mahmoodabad SS, zeidabadi B. Rajabalipour mr. An application of the theory of planned behavior to predict the protective behaviors from urban traffic accidents. Tolooebehdasht. 2023;22(4):28–40.

[4] Shahsavari S, Mohammadi A, Mostafaei S, Zereshki E, Tabatabaei SM, Zhaleh M, et al. Analysis of injuries and deaths from road traffic accidents in iran: bivariate regression approach. BMC Emerg Med. 2022;22(1):130. 35843936 10.1186/s12873-022-00686-6 PMC9290223

[5] Akbari M. Social pathology in Iran; Spatial analysis from the perspective of social geography. Geographical Researches. 2021;36(1):1–11.

[6] Zhao D, Zhong Y, Fu Z, Hou J, Zhao M. A review for the driving behavior recognition methods based on vehicle multisensor information. J Adv Transp. 2022;2022(1):7287511.

[7] Ghaneian MT, Tarfiei A, Ehrampoush MH, Lotfi MH, Namayandeh M, Adamizadeh AR, Keyghobdy N. An epidemiological survey on factors related to traffic accidents in Yazd City, center of Iran (2016–2018). J Occup Health Epidemiol. 2021;10(4):231–8.

[8] Mohan D, Jha A, Chauhan SS. Future of road safety and SDG 3.6 goals in six Indian cities. IATSS Res. 2021;45(1):12–8.

[9] Graneheim UH, Lundman B. Qualitative content analysis in nursing research: concepts, procedures and measures to achieve trustworthiness. Nurse Educ Today. 2004;24(2):105–12. 14769454 10.1016/j.nedt.2003.10.001

[10] Ramos P, Díez E, Pérez K, Rodriguez-Martos A, Brugal MT, Villalbí JR. Young people’s perceptions of traffic injury risks, prevention and enforcement measures: a qualitative study. Accid Anal Prev. 2008;40(4):1313–9. 18606261 10.1016/j.aap.2008.02.001

[11] Hsieh HF, Shannon SE. Three approaches to qualitative content analysis. Qual Health Res. 2005;15(9):1277–88. 16204405 10.1177/1049732305276687

[12] Manning Smith R, Cambiano V, Colbourn T, Collins JH, Graham M, Jewell B, et al. Estimating the health burden of road traffic injuries in Malawi using an individual-based model. Injury Epidemiol. 2022;9(1):21. 10.1186/s40621-022-00386-6 PMC9275162 35821170

[13] Moradi A, Kavousi A, Ameri P, Amjadian M, Vaziri MH. Identifying and prioritizing risk factors involved in motorcyclists’ traffic accidents in Tehran. Archives Trauma Res. 2021;10(3):153–60.

[14] Persia L, Usami DS, De Simone F, Beaumelle VFDL, Yannis G, Laiou A, et al. Management of road infrastructure safety. Transp Res Procedia. 2016;14:3436–45.

[15] Haghani M, Behnood A, Dixit V, Oviedo-Trespalacios O. Road safety research in the context of low-and middle-income countries: macro-scale literature analyses, trends, knowledge gaps and challenges. Saf Sci. 2022;146:105513.

[16] Eisapareh K, Nazari M, Kaveh MH, Cousins R, Mokarami H. Effects of an educational intervention program based on the PRECEDE–PROCEED model for anger management and driving performance of urban taxi drivers: a comparison of traditional and online methods. Saf Sci. 2023;157:1–13.

[17] Ferdosian Z, Morowatisharifabad MA, Rezaeipandari H. Unlicensed motorcycling of high school adolescents in Dehaghan County (Isfahan Province of Iran). Accid Anal Prev. 2015;75:211–6. 25496778 10.1016/j.aap.2014.12.002

[18] Mayhew DR, Simpson HM. The safety value of driver education an training. Inj Prev. 2002;8(suppl 2):ii3–8. 12221024 10.1136/ip.8.suppl_2.ii3 PMC1765489

[19] Senserrick T, McRae D, Rome Ld PPW, Rees P, Williamson A. Enhancing Higher-Order skills education and assessment in a graduated motorcycle licensing system. Safety. 2017;3(2):14.

